# ﻿Two new bamboo-feeding species of the planthopper genus *Bambusicaliscelis* Chen & Zhang, 2011 (Hemiptera, Fulgoromorpha, Caliscelidae) from China

**DOI:** 10.3897/zookeys.1183.110917

**Published:** 2023-11-01

**Authors:** Nian Gong, Xiang-Sheng Chen, Lin Yang

**Affiliations:** 1 Guizhou Provincial Engineering Research Center of Medical Resourceful Healthcare Products, Guiyang Healthcare Vocational University, Guiyang, Guizhou, 550081, China Guiyang Healthcare Vocational University Guiyang China; 2 Institute of Entomology, Guizhou University, Guiyang, Guizhou, 550025, China Guizhou University Guiyang China

**Keywords:** Bamboo, Caliscelini, distribution, Fulgoroidea, morphology, Oriental region, taxonomy

## Abstract

Two new species of the Chinese caliscelid planthopper genus *Bambusicaliscelis* Chen & Zhang, 2011, *B.bibulbus***sp. nov.** from Fujian Province and *B.clavatus***sp. nov.** from Jiangxi Province, are described and illustrated. The genus now known to include six described species. A checklist and identification key to *Bambusicaliscelis* species are provided.

## ﻿Introduction

The caliscelid genus *Bambusicaliscelis* was erected by Chen and Zhang (2011) based on two species, *B.dentis* Chen & Zhang, 2011, and *B.fanjingensis* Chen & Zhang, 2011, from Guizhou Province, southern China. It belongs to the tribe Caliscelini of the subfamily Caliscelinae (Hemiptera, Fulgoroidea, Caliscelidae) (Chen and Zhang 2011). Two species, *B.flavus* Chen & Gong, 2018, and *B.guttatus* Chen & Gong, 2018, were later described from Yunnan and Guangxi Provinces of southern China (Gong et al. 2018). *Bambusicaliscelis* species are similar to one another, but they can be easily distinguished by their male genitalia. All species of the genus are small-bodied (body length ca 3.1–5.1 mm), flightless, and feed on bamboo according to Chen and Zhang (2011) and Gong et al. (2018).

Here, we describe and illustrate two new species, *B.bibulbus* sp. nov. and *B.clavatus* sp. nov., from Fujian and Jiangxi Provinces of southern China and collected on bamboo. Thus, six species are now known in the genus. A checklist and a key based on male genitalia to all known *Bambusicaliscelis* species are provided.

## ﻿Materials and methods

Terminology used for the external morphology and the male genitalia mainly follows Chan and Yang (1994) and Chen and Zhang (2011). The description of the female genitalia mainly follows Bourgoin (1993). Dry specimens were used for the descriptions and illustrations. External morphology was observed under a stereoscopic microscope and characters were measured with an ocular micrometer. All measurements are given in millimeters (mm); body length was measured from the apex of the head to tip of the abdomen in repose. The genital segments of the examined specimens were macerated in 10% NaOH and examined in glycerin jelly using a Leica MZ 12.5 stereomicroscope. Photographs were taken with a KEYENCE VHX-1000 system. Illustrations were scanned with CanoScan LiDE 200 and imported into Adobe Photoshop CS6 for labelling and composition of the figures. The dissected male genitalia are preserved in glycerine in small plastic tubes pinned together with the specimens.

The type specimens and material examined are deposited in the Institute of Entomology, Guizhou University, Guiyang, China (**IEGU**).

## ﻿Taxonomy

### ﻿Order Hemiptera Linnaeus, 1758

**Suborder Fulgoromorpha Evans, 1946**

**Family Caliscelidae Amyot & Audinet-Serville, 1843**

**Subfamily Caliscelinae Amyot & Serville, 1843**

**Tribe Caliscelini Amyot & Serville, 1843**

#### ﻿ Bambusicaliscelis

Taxon classificationAnimaliaHemipteraCaliscelidae

Chen & Zhang, 2011

10F657FC-D0BE-51A1-88A8-7F53984F072F

##### Type species.

*Bambusicaliscelisfanjingensis* Chen & Zhang, 2011, by original designation. *Bambusicaliscelis* Chen & Zhang, 2011: 95; Chen et al. 2014: 157; Gong et al. 2018: 81.

##### Diagnosis.

For the diagnosis of *Bambusicaliscelis*, see Gong et al. (2018: 82). In addition, the hind tibiae has one spine at middle. Spinal formula of hind leg 6 (7)-3-2.

##### Host plant.

Bamboo.

##### Distribution.

Southern China (Guizhou, Yunnan, Guangxi, Jiangxi, Fujian provinces) (Fig. 5).

### ﻿Checklist of species of *Bambusicaliscelis* Chen & Zhang, 2011

*B.dentis* Chen & Zhang, 2011; China (Guizhou).

*B.fanjingensis* Chen & Zhang, 2011; China (Guizhou).

*B.flavus* Chen & Gong, 2018; China (Yunnan).

*B.guttatus* Chen & Gong, 2018; China (Guangxi).

*B.bibulbus* Gong & Yang, sp. nov.; China (Fujian).

*B.clavatus* Gong & Yang, sp. nov.; China (Jiangxi).

### ﻿Key to species of genus *Bambusicaliscelis* (modified from Gong et al. 2018) (males)

**Table:** 

1	The ratio of vertex base width to median length is less than 2; phallus without any teeth-like processes	**2**
–	The ratio of vertex base width to median length is more than 2; phallus with 2–3 teeth-like processes (Chen and Zhang 2011: figs 19, 20)	***B.dentis***
2	Forewing yellow; pygofer in lateral view (Gong et al. 2018: fig. 8) with posterior margin sinuate	***B.flavus***
–	Forewing brown; pygofer in lateral view with posterior margin concave in the middle	**3**
3	Forewing with one large marking near apical margin	***B.guttatus***
–	Forewing without marking near apical margin	**4**
4	Forewing with one pale yellow marking near base	***B.bibulbus* sp. nov.**
–	Forewing without any marking near base	**5**
5	Spinal formula of hind leg 6-3-2; pygofer in posterior view (Chen and Zhang 2011: fig. 7) ventral margin with short, broad medioventral process, lobe-like	***B.fanjingensis***
–	Spinal formula of hind leg 7-3-2; pygofer in posterior view (Fig. 4(H)) ventral margin with claviform medioventral process	***B.clavatus* sp. nov.**

#### ﻿ Bambusicaliscelis bibulbus sp. nov.

Taxon classificationAnimaliaHemipteraCaliscelidae

CCFECB1B-30D8-50E8-96DC-3C59340E1426

https://zoobank.org/BE41F1DA-FB57-437A-B1C3-CD629F64FA39

Figs 1 , 2 , 3

##### Description.

***Measurements*.** Body length (from apex of vertex to tip of abdomen): male 3.9–4.1 mm (*N* = 3), female 4.9–5.1 mm (*N* = 4); forewing length: male 1.7–1.8 mm (*N* = 3), female 1.9–2.1 mm (*N* = 4).

***Colouration*. Male**: body mainly brown; longitudinal stripe from apex of vertex to tip of abdomen pale yellow. Frons dark brown, with small, yellowish-white tubercules between lateral and sublateral carinae. Clypeus dark brown. Eyes yellowish brown. Forewing brown, with one pale yellow marking near base. Abdominal 4^th^ and 5^th^ pleuron pale yellow. Female: body mainly brown but some green; longitudinal stripe from apex of vertex to tip of abdomen inconspicuous and pale yellow. Eyes and forewing brown.

**Figure 1.**
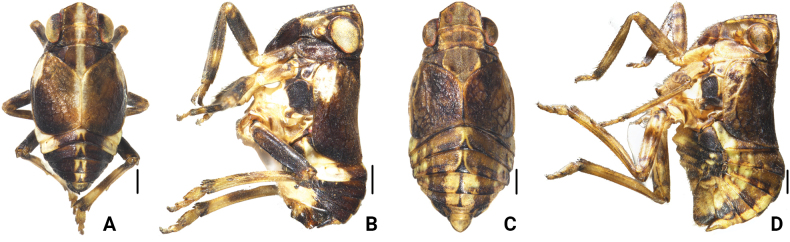
*Bambusicaliscelisbibulbus* sp. nov. **A** male habitus, dorsal view **B** male habitus, lateral view **C** female habitus, dorsal view **D** female habitus, lateral view. Scale bars: 0.5 mm (**A–D**).

***Head and thorax*.** Vertex with anterior margin subtruncate; width of vertex, including eyes, as long as pronotum. Vertex at middle 0.6 times wider than width at base. Frons 1.1 times longer in middle than at widest part; sublateral carinae slightly keeled; median carina distinct; areas between sublateral carinae and lateral carinae slightly depressed. Pronotum as long as vertex. Mesonotum at middle 0.8 times as long as vertex and pronotum together, 3-carinate, median carina weak. Spinal formula of hind leg 7-3-2.

**Figure 2.**
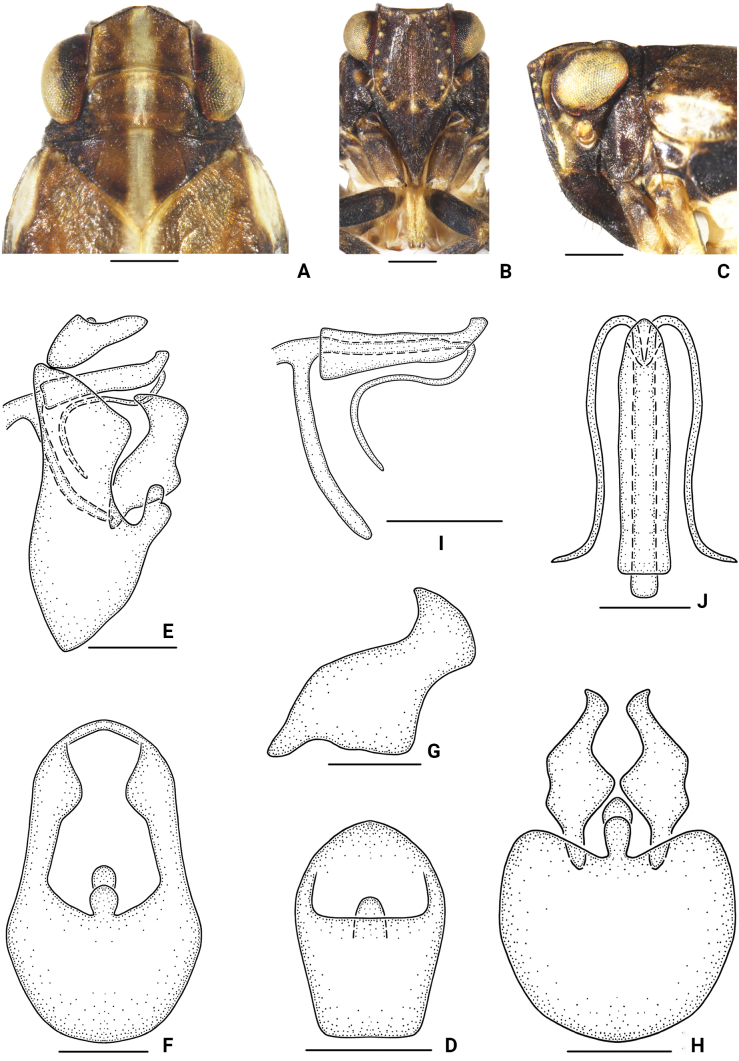
*Bambusicaliscelisbibulbus* sp. nov., male **A** head and thorax, dorsal view **B** face **C** head and thorax, lateral view **D** anal segment, dorsal view **E** genitalia, lateral view **F** pygofer, posterior view **G** genital styles, lateral view **H** pygofer and genital styles, ventral view **I** aedeagus, lateral view **J** aedeagus, dorsal view. Scale bars: 0.5 mm (**A–C, E, F, H**); 0.3 mm (**D, G, I, J**).

***Male genitalia*.** Anal segment in dorsal view 1.5 times longer at middle than at widest part; apical margin roundly convex, broadening apically, and broadest at apical third; dorsal margin in lateral view slightly convex, widest at apical half, thence constricted, ventral margin slightly concave in the middle. Pygofer in lateral view with posterior margin with upper half roundly convex, lower half heart-shaped, convex, and ventral margin strongly oblique; in posterior view 1.7 times as long as widest part; in ventral view, posterior margin with a double-droplet-shaped medioventral process. Genital style in lateral view with basal half broad, apical third narrowest; apical margin slightly convex; a finger-like process apically arises from dorsal margin, slightly dorsally curved; genital style in ventral view long, median portion broad. Phallobase tubular, with broad base, narrowing apically, apex ventrally divided into double petals. Aedeagus double-tubular, much more slender and longer, encircled in phallobase, reflexed basad at level of apex of phallobase, tapering apically.

**Figure 3.**
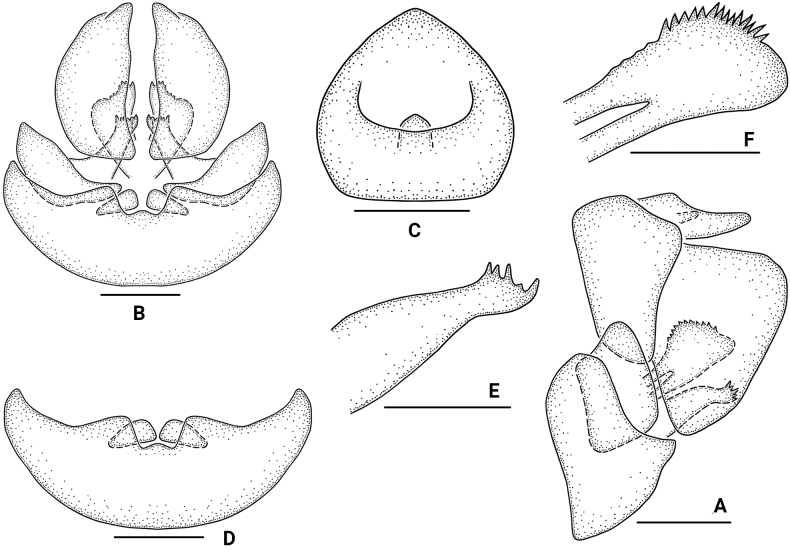
*Bambusicaliscelisbibulbus* sp. nov., female **A** genitalia, lateral view **B** genitalia, ventral view **C** anal segment, dorsal view **D** abdominal sternite VII, ventral view **E** gonapophysis VIII, lateral view **F** gonapophysis IX, lateral view. Scale bars: 0.5 mm (**A, B, D**); 0.3 mm (**C, E, F**).

***Female genitalia*.** Anal segment in dorsal view 0.9 times longer at middle than at widest part; apex narrowed; apical margins convex; anal pore located in the middle, in lateral view small, narrowing apically. Abdominal sternite VII in ventral view with width 3.6 times as long as length; posterior margin strongly trapezoidal concave, behind the posterior margin with two small, triangular ossification flakes. Gonapophysis VIII (first valvula) elongate, with five spines at apical margin. Gonapophysis IX (second valvula) with two symmetrical lobes; each lobe with many spines at dorsal margin. Gonoplac (third valvula) with outer surface shagreened; in lateral view broad, nearly triangular.

##### Host plant.

Bamboo.

##### Distribution.

China (Fujian Province) (Fig. 5).

##### Type materials.

***Holotype***: ♂, China: Fujian Province, Jianan County, Nanping Park, 2019-VIII-10, Zhicheng Zhou. ***Paratypes***, 5♂♂, 7♀♀: data same as holotype, Xiaoya Wang and Yongjin Sui.

##### Etymology.

The specific name is derived from a combination of the Latin “*bi*-” (two) and “*bulbus*” (bulb), referring to the shape of the medioventral processes on the posterior margin of the pygofer.

##### Remarks.

This new species is closely related to *B.fanjingensis* Chen & Zhang, 2011, but differs in the following: 1) forewing with one pale-yellow mark near base (without any mark in *B.fanjingensis*); 2) posterior margin of pygofer with a strongly medioventral process which is double-droplet-shaped (with only a slightly medioventral process in *B.fanjingensis*); 3) genital style in lateral view long and narrow, and ventral margin roundly concave in the middle (genital style with median portion broad and large, and ventral margin roundly convex in *B.fanjingensis*).

#### ﻿ Bambusicaliscelis clavatus sp. nov.

Taxon classificationAnimaliaHemipteraCaliscelidae

6531CE12-D671-5638-A4B5-ADA1C56C6861

https://zoobank.org/D9F25415-6E02-4120-A4E4-D00F533FA077

Fig. 4

##### Description.

***Measurements*.** Body length (from apex of vertex to tip of abdomen): male 4.3 mm; forewing length: male 1.8 mm.

***Coloration*.** Body mainly brown; longitudinal stripe from apex of vertex to tip of abdomen pale yellow. Frons brown with small, yellowish-white tubercules between lateral and sublateral carinae. Clypeus dark brown. Eyes yellowish brown. Pronotum and mesonotum lateral areas with several yellowish-brown sensory pits. Forewing brown to dark brown. Abdominal 4^th^ and 5^th^ pleuron pale yellow.

***Head and thorax*.** Vertex with anterior margin subtruncated, width of vertex including eyes as long as pronotum. Vertex at middle 0.6 times wider than width at base. Frons 0.9 times wider at middle than widest part; sublateral carinae slightly keeled; median carina distinct; areas between sublateral carinae and lateral carinae slightly depressed. Pronotum as long as vertex. Mesonotum 0.8 times as long as vertex and pronotum together in middle line, 3-carinate; median carina weak. Spinal formula of hind leg 7-3-2.

***Male genitalia*.** Anal segment in dorsal view 1.4 times longer at middle than at widest part, narrowing apically, with apical margin roundly convex; in lateral view dorsal margin slightly convex; ventral margin slightly concave in middle, base half as broad, apex half as narrow. Pygofer in lateral view with posterior margin with upper half roundly convex, lower half strongly quadrangular convex, ventral margin strongly oblique; in posterior view 2.1 times longer than at its widest part; in ventral view, posterior margin with a claviform medioventral process. Genital style in lateral view large and broad, ventral margin convex, dorsal margin sinuate, apical margin slightly convex; finger-like process apically arising from dorsal margin, slightly dorsally curved, narrowing apically; in ventral view long, median portion broad. Phallobase tubular; apex ventrally divided into double petals; ventral margin of distal third resembles a finger process. Aedeagus double-tubular, much more slender and longer, encircled in phallobase, reflexed dorsad at level of apical third of phallobase, proximal ventrally curved toward apex, tapering apically.

**Figure 4.**
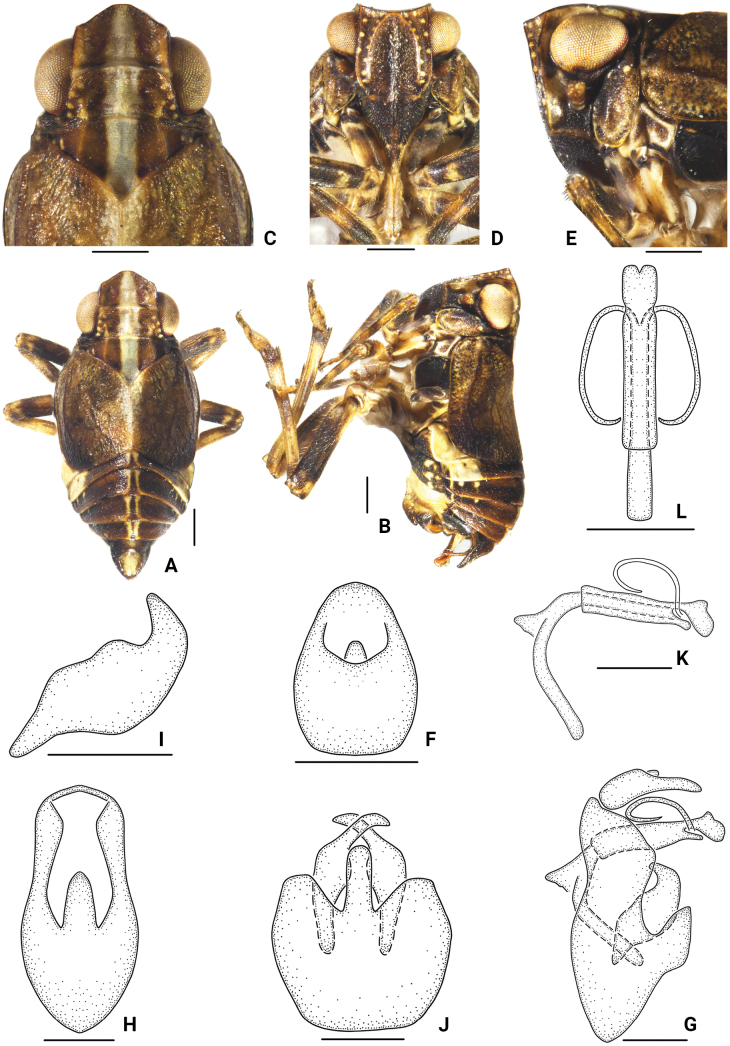
*Bambusicaliscelisclavatus* sp. nov., male **A** habitus, dorsal view **B** habitus, lateral view **C** head and thorax, dorsal view **D** face **E** head and thorax, lateral view **F** anal segment, dorsal view **G** male genitalia, lateral view **H** pygofer, posterior view **I** genital styles, lateral view **J** pygofer and genital styles, ventral view **K** aedeagus, lateral view **L** aedeagus, dorsal view. Scale bars: 0.5 mm (**A–E, G, H, J**); 0.3 mm (**F, I, K, L**).

##### Host plant.

Bamboo.

##### Distribution.

**Figure 5.**
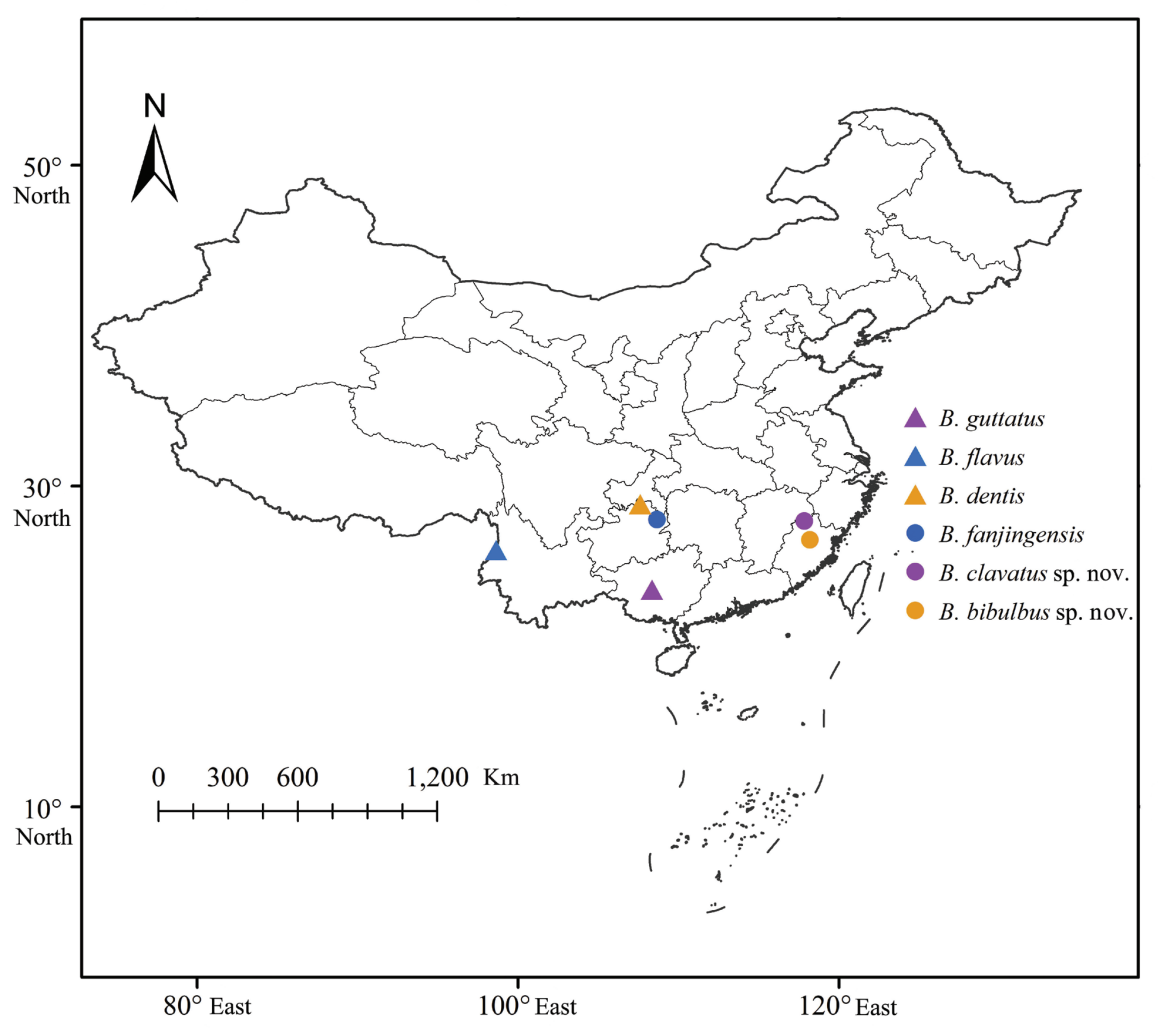
Geographic distributions of species of *Bambusicaliscelis* Chen & Zhang, 2011.

China (Jiangxi Province) (Fig. 5).

##### Type materials.

***Holotype***: ♂, China: Jiangxi Province, Qianshan County, Wuyi Mountains, 2018-VIII-19, Xiangsheng Chen. ***Paratype***, 1♂: data same as holotype, 2013-VIII-3, Jiankun Long.

##### Etymology.

The specific name is derived from the Latin words “*clava*” (a staff or club), referring to the shape of the claviform medioventral process.

##### Remarks.

This new species is closely related to *B.fanjingensis* Chen & Zhang, 2011, but differs by the following: 1) spinal formula of hind leg 7-3-2 (spinal formula of hind leg 6-3-2 in *B.fanjingensis*); 2) posterior margin of pygofer with a strongly claviform medioventral process (with a slightly medioventral process in *B.fanjingensis*); 3) aedeagus reflexed dorsad (reflexed ventrad in *B.fanjingensis*).

## ﻿Discussion

Based on published information as well as available specimens, we have found that the phallobase in all species of is of the “closed-tube” type. Thus, by this character, *Bambusicaliscelis* may be considered one of the most primitive members of Caliscelini. Compared to the “open-tube” type found in other Peltonotellini (Emeljanov 2008: figs 2, 3) and Caliscelini (Gnezdilov and Bourgoin 2009: figs 63–65), this may suggest a primitive (ancestral) condition.

According to characters of their genitalia, *B.fanjingensis*, *B.guttatus*, *B.flavus*, and *B.dentis* are similar in having the genital style with a narrow capitulum. *Bambusicaliscelisfanjingensis*, *B.guttatus*, *B.dentis*, and *B.bibulbus* sp. nov. all share a ventrally reflexed aedeagus. *Bambusicaliscelisfanjingensis*, *B.guttatus*, *B.bibulbus* sp. nov., and *B.clavatus* sp. nov. have the ventral margin of the pygofer in posterior view with a medioventral process. In *B.dentis*, *B.fanjingensis*, *B.guttatus*, *B.bibulbus* sp. nov., and *B.clavatus* sp. nov. the pygofer in lateral view has the medio-posterior margin concave.

## Supplementary Material

XML Treatment for Bambusicaliscelis

XML Treatment for Bambusicaliscelis bibulbus

XML Treatment for Bambusicaliscelis clavatus

## ﻿Additional information

### Author ORCIDs

Nian Gong https://orcid.org/0000-0002-8878-5337

Xiang-Sheng Chen https://orcid.org/0000-0001-9801-0343

Lin Yang https://orcid.org/0000-0002-7841-5156

### Data availability

All of the data that support the findings of this study are available in the main text.
